# Incretin accelerates platelet-derived growth factor-BB-induced osteoblast migration via protein kinase A: The upregulation of p38 MAP kinase

**DOI:** 10.1038/s41598-020-59392-7

**Published:** 2020-02-11

**Authors:** Tetsu Kawabata, Haruhiko Tokuda, Gen Kuroyanagi, Kazuhiko Fujita, Go Sakai, Woo Kim, Rie Matsushima-Nishiwaki, Hiroki Iida, Ken-ichiro Yata, Shujie Wang, Akira Mizoguchi, Takanobu Otsuka, Osamu Kozawa

**Affiliations:** 1 Department of Pharmacology, Gifu University Graduate School of Medicine, Gifu, 501-1194 Japan; 2 Department of Anesthesiology and Pain Medicine, Gifu University Graduate School of Medicine, Gifu, 501-1194 Japan; 3 Department of Orthopedic Surgery, Nagoya City University Graduate School of Medical Sciences, Nagoya, 467-8601 Japan; 4 Department of Rehabilitation Medicine, Nagoya City University Graduate School of Medical Sciences, Nagoya, 467-8601 Japan; 5 Department of Orthopedic Surgery, Toyokawa City Hospital, Toyokawa, 442-8561 Japan; 6 Department of Clinical Laboratory/Medical Genome Center Biobank, National Center for Geriatrics and Gerontology, Obu, 474-8511 Japan; 7 Department of Neurology, Graduate School of Medicine, Mie University, Tsu, 514-8507 Japan; 8 Deaprtment of Neural Regeneration and Cell Communication, Graduate School of Medicine, Mie University, Tsu, 514-8507 Japan

**Keywords:** Cell biology, Endocrinology

## Abstract

Incretins, including glucagon-like peptide-1 (GLP-1) and glucose-dependent insulinotropic polypeptide (GIP), secreted from enteroendocrine cells after food ingestion, are currently recognized to regulate glucose metabolism through insulin secretion. We previously demonstrated that platelet-derived growth factor-BB (PDGF-BB) induces the migration of osteoblast-like MC3T3-E1 cells through mitogen-activated protein (MAP) kinases, including p38 MAP kinase. In the present study, we investigated whether or not incretins affect the osteoblast migration. The PDGF-BB-induced cell migration was significantly reinforced by GLP-1, GIP or cAMP analogues in MC3T3-E1 cells and normal human osteoblasts. The upregulated migration by GLP-1 or cAMP analogues was suppressed by H89, an inhibitor of protein kinase A. The amplification by GLP-1 of migration induced by PDGF-BB was almost completely reduced by SB203580, a p38 MAP kinase inhibitor in MC3T3-E1 cells and normal human osteoblasts. In addition, GIP markedly strengthened the PDGF-BB-induced phosphorylation of p38 MAP kinase. Exendin-4, a GLP-1 analogue, induced Rho A expression and its translocation from cytoplasm to plasma membranes in osteoblasts at the epiphyseal lines of developing mouse femurs *in vivo*. These results strongly suggest that incretins accelerates the PDGF-BB-induced migration of osteoblasts via protein kinase A, and the up-regulation of p38 MAP kinase is involved in this acceleration. Our findings may highlight the novel potential of incretins to bone physiology and therapeutic strategy against bone repair.

## Introduction

Bone metabolism is tightly controlled by osteoblasts and osteoclasts^1,2^. The former cells are responsible for bone formation and the latter for bone resorption. The tissue of bone is constantly regenerated through a remodeling process^1^. In the bone remodeling process, osteoclastic bone resorption is the first step, following bone formation by osteoblasts, and appropriate bone volume is sophisticatedly maintained by the balanced activity of osteoclasts and osteoblasts.

Bone remodeling impairment elicits metabolic bone diseases, including osteoporosis. The migration of osteoblasts to sites of resorption by osteoclasts is recognized critical for many physiologically essential processes in bone metabolism, such as the responses to load and remodeling^1,3,4^. The osteoblast migration importantly participates in the pathological processes including fracture repair and metastasis of tumor^5^.

Various humoral factors, such as platelet-derived growth factor (PDGF), play crucial roles in bone metabolism^3,4^. PDGF is an important mitogen in connective tissue cells, including osteoblasts^6,7^. Embedded PDGF in the matrix of bone plays an important role in the process of bone fracture healing during bone metabolism^8^. The administration of PDGF-BB reportedly facilitates the healing from fracture observed in geriatric osteoporotic rats^9^. It has recently been shown that PDGF-BB promotes the human osteoblasts migration^10^. We demonstrated that the activation of p44/p42 mitogen-activated protein (MAP) kinase, p38 MAP kinase and stress-activated protein kinase/c-*Jun* N-terminal kinase (SAPK/JNK) are involved in the migration of osteoblast-like MC3T3-E1 cells stimulated by PDGF-BB^11,12^. However, the exact mechanism underlying the PDGF-induced migration of osteoblasts remains to be clarified.

Incretin is a hormone released from the small intestinal enteroendocrine cells in response to oral intake of food^13^. Incretin stimulates the secretion of insulin from pancreatic islet β cells and inhibits that of glucagon from pancreatic α cells, resulting in the suppression of the serum glucose level^13,14^. Glucagon-like peptide-1 (GLP-1) and glucose-dependent insulinotropic polypeptide (GIP) are generally recognized as incretins^13^. A GLP-1 receptor agonist and a dipeptidyl peptidase-IV inhibitor are presently used in clinical setting as medications for patients with type 2 diabetes mellitus^14^. The insulinotropic effects of GLP-1 and GIP are exerted via specific guanine nucleotide-binding protein (G-protein)-coupled receptors which are expressed on the surface of pancreatic β cells^15^.

It is generally recognized that the binding of incretin to its receptors causes the activation of the adenylyl cyclase/cAMP/protein kinase A pathway, leading to insulin secretion^13^. Accumulating evidence indicates that incretin affects the cell functions of not only pancreatic cells but also mesenchymal cells such as osteoblasts and adipocytes^15,16^. Regarding the effects of incretin on bone, it has been shown that GIP increases the bone mineral density in ovariectomized rats^17^. An increased number of osteoclasts and accelerated bone resorption are reportedly observed in GLP-1 receptor-deficient mice which suffer from osteoporosis^18^. In osteoblasts, GIP stimulates both the collagen type I expression and the activity of alkaline phosphatase in osteoblasts^19^. In addition, GLP-1 is reported to induce the differentiation of osteoblasts^20^. However, the details behind the effects of incretin on bone metabolism have not yet been precisely elucidated.

Given the reported roles of incretin in mesenchymal cells, we hypothesized that incretin might be involved in osteoblast migration. In addition, the intracellular translocation of Rho A, a major small G protein regulating cell motility and migration through cytoskeletal reorganization via myosin light chain and actin polymerization, is recognized as an indicator of migration onset^21^. We herein investigated the effects of GLP-1 and GIP on the PDGF-BB-induced migration of osteoblast-like clonal MC3T3-E1 cells. We demonstrated that incretin amplifies the PDGF-BB-induced migration of these cells via protein kinase A and that this amplification was mediated via p38 MAP kinase activation at least in part. We also showed the translocation of Rho A induced by incretin analogues in osteoblasts *in vivo*, which supports the physiological role of incretin in the migration.

## Materials and Methods

### Materials

PDGF-BB was purchased form R&D System, Inc. (Minneapolis, MN, USA). GLP-1 and GIP were obtained from Peptide Institute, Inc. (Osaka, Japan). 8-Bromo cAMP, PD98059, SP600125 or SB203580 were obtained from Calbiochem-Novabiochem Co. (La Jolla, CA, USA). Dibutyryl (Bt2) cAMP was obtained from Sigma-Aldrich Co. (St. Louis, MO, USA). H89 was obtained from Seikagaku Co. (Tokyo, Japan). Phospho-specific cAMP response element-binding protein (CREB) antibodies and phospho-specific p38 MAP kinase antibodies were purchased from Cell Signaling Technology, Inc. (Beverly, MA, USA). Glyceraldehyde-3-phosphate dehydrogenase (GAPDH) antibodies, anti-osteocalcin mouse antibody and anti-Rho-A (26C4) mouse monoclonal antibody were obtained from Santa Cruz Biotechnology, Inc. (Santa Cruz, CA, USA). An ECL Western blotting detection system was obtained from GE Healthcare Life Sciences (Chalfront, UK). Exendin-4 was obtained from Abcam (Cambridge, UK). Other materials and chemicals were obtained from commercial sources. H89, PD98059, SP600125 or SB203580 were dissolved in dimethyl sulfoxide. The maximum concentration of dimethyl sulfoxide was 0.3%, which did not affect the cell migration assay or detection of the protein level using Western blotting^22^.

### Cell migration assay

Cloned osteoblast-like MC3T3-E1 cells that have been derived from newborn mouse calvaria^23^ were maintained as previously described^24^. In brief, the cells were cultured in α-minimum essential medium (α-MEM) supplemented with 10% fetal bovine serum (FBS) in a 5% CO_2_ humidified incubator at 37 °C. Normal human osteoblasts (NHOsts) isolated from human tissue obtained under “informed consent” were obtained from CAMBREX (Charles, IA, USA) and cultured under conditions similar to those for MC3T3-E1 cells, as previously described^25^. The migration of MC3T3-E1 cells or NHOsts was analyzed by a wound-healing assay as previously described^22^. In brief, the cells were seeded at 10 × 10^4^ cells/well into an Ibidi Culture-Insert 2 Well (Ibidi, Martinsried, Germany) with a 500-μm margin from the side of the well and cultured for 24 h. After removing the insert and performing pretreatment with GLP-1, GIP, 8-bromo cAMP or Bt2 cAMP for 60 min, the cells were stimulated by PDGF-BB for the indicated periods. Cells were photographed with an EOS Kiss X4 digital camera (Canon, Tokyo, Japan) connected to a CK40 culture microscope (Olympus Optical Co. Ltd., Tokyo, Japan), and the area of migrated cells was calculated by the ImageJ software program (version 1.48; NIH, Bethesda, MD, USA). When indicated, the cells were incubated with H89, PD98059, SP600125 or SB203580 for 60 min prior to pretreatment.

### Western blot analyses

MC3T3-E1 cells were seeded into 90-mm diameter dishes (2 × 10^5^ cells/dish) in α-MEM containing 10% FBS. After 5 days, the medium was exchanged for α-MEM containing 0.3% FBS. After 48 h, the cells were used for a Western blot analysis as previously described^22^. The stimulation with GLP-1 or PDGF-BB to the cultured cells were performed in 1 ml of 0.3% FBS-containing α-MEM for a variety of times indicated. In the indicated experiments, the pretreatment with H89 or GIP to the cells were performed for 60 min prior to the stimulation. The cells were then lysed, homogenized and sonicated in a lysis solution composed of 62.5 mM Tris/HCl, pH 6.8, 2% sodium dodecyl sulfate (SDS), 50 mM dithiothreitol and 10% glycerol^22^. SDS-polyacrylamide gel electrophoresis (PAGE) was performed by the method of Laemmli^26^ in 10% polyacrylamide gels^22^. The protein was fractionated and transferred onto an Immun-Blot PVDF membrane (Bio-Rad, Hercules, CA, USA)^22^. The membranes were blocked with 5% fat-free dry milk in Tris-buffered saline-Tween (TBS-T; 20 mM Tris-HCl, pH 7.6, 137 mM NaCl, 0.1% Tween 20) for 1 h before incubation with primary antibodies^22^. Western blot analyses were performed as described previously^27^ using phospho-specific CREB antibodies (1:1000), GAPDH antibodies (1:1000) or phospho-specific p38 MAP kinase antibodies (1:20000) as primary antibodies with the secondary antibodies, peroxidase-labeled antibodies raised in goat-anti-rabbit IgG (1:1000) (KPL, Inc., Gaithersburg, MD, USA). The primary and secondary antibodies were diluted with 5% fat-free dry milk in TBS-T^22^. The peroxidase activity on the PVDF sheet was visualized on an X-ray film using the ECL Western blotting detection system^22^.

### Densitometric analyses

Band densities were measured using a scanner and the ImageJ software program (NIH) as previously described^22^. The phosphorylated levels were calculated as follows: the signal intensity of each phosphorylation subtracted the background signal was normalized to the respective GAPDH intensity and plotted to indicate the fold increase compared to the control cells without stimulation^22^.

### *In vivo* experiments

This study was approved by the Animal Research Committee of Mie University. Twelve male C57BL/6 mice at postnatal day 10 were used in the *in vivo* experiments (Japan SLC, Inc., Shizuoka, Japan). All procedures were performed in accordance with the guidelines for animal experimentation outlined by the ethics committee of Mie University.

### Immunohistochemical analyses of Rho A in osteoblasts in response to exendin-4

Twelve male mice went without food for 8 h before the tests. Exendin-4, a GLP-1 analogue^28^, was intraperitoneally administered at 100 ng/g body weight. The mice with or without exendin-4 administration were perfused with a fixation solution containing 4% paraformaldehyde 1 and 2 h after the administration. The samples were immediately frozen into OCT compound (Sakura Finetek, Tokyo, Japan), and 14-μm-thick frozen sections containing the epiphyseal lines of the femurs were blocked with 0.1 M phosphate buffer (pH 7.4) containing 4% Block Ace (DS Pharma Biomedical), 0.02% saponin and protease cocktail. The samples were incubated at room temperature (RT) for 20 min before being incubated either with anti-osteocalcin mouse antibody (1:500), an osteoblast marker, or with anti-Rho A (26C4), a mouse monoclonal antibody (1:500), at 4 °C overnight, and with the respective secondary antibodies for 2 h at RT with or without phalloidin and DRAQ5(1:2000), to visualize actin filaments and nuclei, respectively. Immunohistochemical and immunofluorescence signals were photographed with a confocal laser scanning microscopy (FV3000; Olympus).

### Statistical analyses

We adopted a parametric analysis approach, and the data were evaluated by an analysis of variance (ANOVA) followed by Bonferroni’s method for multiple comparisons between pairs, as previously described^22^. The statistical significance level was set to p < 0.05. We used nine samples (three wells from three different *in vitro* experiments) for the analysis. All data were presented as the mean ± standard error of the mean of triplicate determinations from thrice-repeated measurements^22^.

## Results

### Effects of GLP-1 or GIP on the PDGF-BB-induced migration of MC3T3-E1 cells and NHOsts

**Figure 1:**
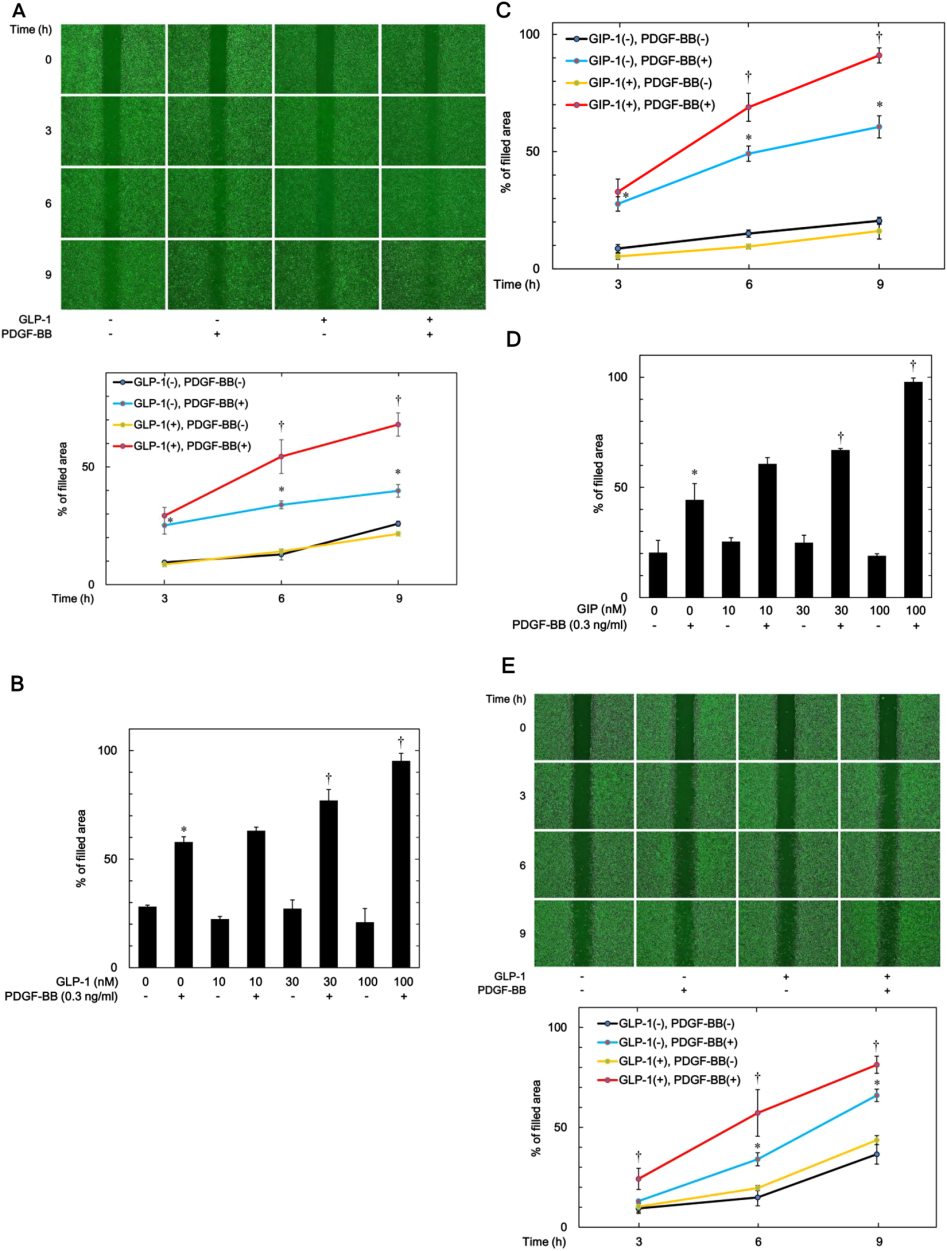
Effect of GLP-1 and GIP on the PDGF-BB-induced migration of MC3T3-E1 cells and NHOsts. (**A**) MC3T3-E1 cells were pretreated with 100 nM of GLP-1 or vehicle for 60 min and then stimulated by 0.3 ng/ml of PDGF-BB or vehicle for the indicated periods. The cells were photographed before PDGF-BB-stimulation (0 h) and 3, 6 or 9 h later (upper panel), and the area of the migrated cells was measured (lower graph). The black line indicates the control value without PDGF-BB stimulation. The blue line indicates the value of PDGF-BB alone. The yellow line indicates the value of GLP-1-treated cells without PDGF-BB stimulation. The red line indicates the value of GLP-1-treated cells with PDGF-BB stimulation. *p < 0.05 compared to the value of the control cells without PDGF-BB stimulation. ^†^*p* < 0.05 com*p*ared to the value of PDGF-BB alone. (**B**) MC3T3-E1 cells were pretreated with various doses of GLP-1 for 60 min and then stimulated by 0.3 ng/ml of PDGF-BB or vehicle for 9 h. The area of the migrated cells was measured. *p < 0.05 compared to the value of the control cells without PDGF-BB stimulation. ^†^*p* < 0.05 compared to the value of PDGF-BB alone. (**C**) MC3T3-E1 cells were pretreated with 100 nM of GIP or vehicle for 60 min and then stimulated by 0.3 ng/ml of PDGF-BB or vehicle for the indicated periods. The area of the migrated cells was measured. The black line indicates the value of control without PDGF-BB stimulation. The blue line indicates the value of PDGF-BB alone. The yellow line indicates the value of GIP-treated cells without PDGF-BB stimulation. The red line indicates the value of GIP-treated cells with PDGF-BB stimulation. *p < 0.05 compared to the value of the control cells without PDGF-BB stimulation. ^†^*p* < 0.05 compared to the value of PDGF-BB alone. (**D**) MC3T3-E1 cells were pretreated with various doses of GIP for 60 min and then stimulated by 0.3 ng/ml of PDGF-BB or vehicle for 9 h. The area of the migrated cells was measured. *p < 0.05 compared to the value of the control cells without PDGF-BB stimulation. ^†^*p* < 0.05 compared to the value of PDGF-BB alone. (**E**) NHOsts were pretreated with 100 nM of GLP-1 or vehicle for 60 min and then stimulated by 0.3 ng/ml of PDGF-BB or vehicle for the indicated periods. The cells were photographed before PDGF-BB-stimulation (0 h) and 3, 6 or 9 h later (upper panel), and the area of the migrated cells was measured (lower graph). The black line indicates the value of control without PDGF-BB stimulation. The blue line indicates the value of PDGF-BB alone. The yellow line indicates the value of GLP-1-treated cells without PDGF-BB stimulation. The red line indicates the value of GLP-1-treated cells with PDGF-BB stimulation. *p < 0.05 compared to the value of the control cells without PDGF-BB stimulation. ^†^*p* < 0.05 compared to the value of PDGF-BB alone.

In our recent study^12^, we found that PDGF-BB significantly stimulated the migration of clonal osteoblast-like MC3T3-E1 cells, consistent with a previous report^10^. We first examined the effect of GLP-1, an incretin, on the PDGF-BB-induced migration of MC3T3-E1 cells. GLP-1, which alone had no effect on the cell migration, markedly enhanced the migration induced by PDGF-BB as assessed by a wound-healing assay in a time-dependent manner (Fig. 1A). The significant amplification by GLP-1 of the cell migration was observed after 6 h (Fig. 1A). GLP-1 dose-dependently augmented the PDGF-BB-induced migration of MC3T3-E1 cells over the range 10 and 100 nM (Fig. 1B). GLP-1 (100 nM) caused a 1.26 ± 0.06-fold change in the PDGF-BB effect (p = 0.009).

In addition, GIP, another incretin, also time-dependently upregulated the PDGF-BB-induced migration of MC3T3-E1 cells (Fig. 1C). The amplifying effect of GIP on the cell migration was dose-dependent over the range 10 and 100 nM (Fig. 1D). GIP (100 nM) caused a 2.38 ± 0.57-fold change in the PDGF-BB effect (p = 0.002).

Similar to the findings with MC3T3-E1 cells, the PDGF-BB-induced migration of NHOsts was significantly accelerated by GLP-1 at 6 and 9 h after PDGF-BB-stimulation (Fig. 1E). We found no significant difference between the control group and the PDGF-BB-induced group in the migration of NHOsts at 3 h. It seems likely that the effect of PDGF-BB on migration of NHOsts requires more than 3 h to manifest, which is longer than that required for MC3T3-E1 cells.

### Effects of 8-bromo cAMP or Bt2 cAMP on the PDGF-BB-induced migration of MC3T3-E1 cells and NHOsts

**Figure 2:**
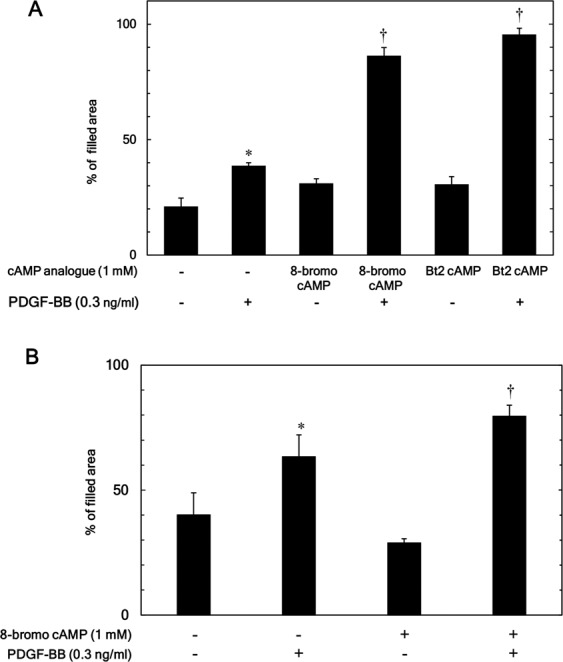
Effects of 8-bromo cAMP or Bt2 cAMP on the PDGF-BB-induced migration of MC3T3-E1 cells and NHOsts. (**A**) MC3T3-E1 cells or (**B**) NHOsts were pretreated with 1 mM 8-bromo cAMP, 1 mM Bt2 cAMP or vehicle for 60 min and then stimulated by 0.3 ng/ml of PDGF-BB or vehicle for 9 h. The area of the migrated cells was measured. *p < 0.05 compared to the value of the control cells without PDGF-BB stimulation. ^†^*p* < 0.05 com*p*ared to the value of PDGF-BB alone.

Evidence is accumulating that the adenylyl cyclase/cAMP/protein kinase A pathway is a main intracellular signaling pathway of incretin^13^. The incretin-induced activation of the signaling pathway reportedly leads to the activation of cAMP response element-binding protein (CREB), a transcriptional factor^29^. We previously found that GLP-1 actually stimulates the activation of CREB in MC3T3-E1 cells^30^. To investigate whether or not the adenylyl cyclase/cAMP/protein kinase A pathway is involved in the enhancement by incretin in MC3T3-E1 cells, we examined the effects of cAMP analogues on the PDGF-BB-induced cell migration. 8-Bromo cAMP (1 mM), a cell-membrane permeable analogue of cAMP^31^, significantly amplified the cell migration induced by PDGF-BB (Fig. 2A). In addition, the PDGF-BB-induced cell migration was markedly augmented by Bt2 cAMP (1 mM), another cell-membrane-permeable cAMP analogue^31^ (Fig. 2A). 8-Bromo cAMP and Bt2 cAMP caused a 2.86 ± 0.59- (p = 0.002), and 3.39 ± 0.62-fold change (p = 0.00004), respectively, in the PDGF-BB effect. We also found that 8-bromo cAMP significantly up-regulated the PDGF-BB-induced migration of NHOsts (Fig. 2B).

### Effects of H89 on the amplification by Bt2 cAMP or GLP-1 of the PDGF-BB-induced migration of MC3T3-E1 cells

**Figure 3:**
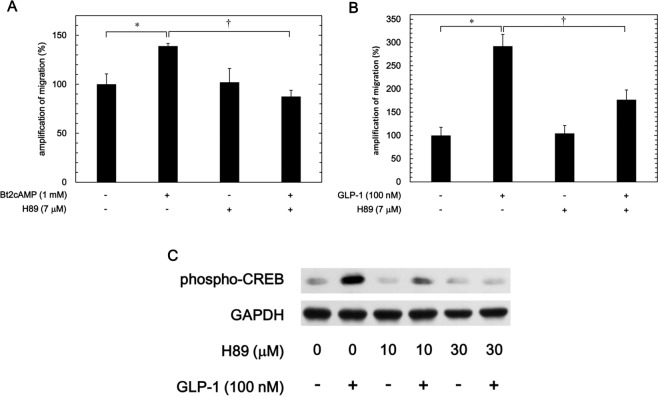
Effects of H89 on the amplification by Bt2 cAMP or GLP-1 of the PDGF-BB-induced migration and GLP-1-induced phosphorylation of CREB in MC3T3-E1 cells. MC3T3-E1 cells were incubated with 7 μM of H89 for 60 min and subsequently pretreated with 1 mM of Bt2 cAMP (**A**), 100 nM of GLP-1 (**B**) or vehicle for 60 min. The cells were then stimulated by 0.3 ng/ml of PDGF-BB or vehicle for 9 h. The area of the migrated cells was measured, and the PDGF-BB-induced increase in the filled area without Bt2 cAMP (**A**) or without GLP-1 (**B**) was presented as 100%. *p < 0.05 compared to the value without Bt2 cAMP (**A**) or without GLP-1 (**B**). ^†^*p* < 0.05 com*p*ared to the value without H89. (**C**) MC3T3-E1 cells were pretreated with various doses of H89 for 60 min and then stimulated by 100 nM of GLP-1 or vehicle for 3 min. The extracts of cells were subjected to SDS-PAGE with subsequent Western blot analyses using antibodies against phospho-specific CREB or GAPDH.

We found that H89, an inhibitor of protein kinase A^32^, which failed to affect the migration by PDGF-BB alone, markedly reduced the amplification by Bt2 cAMP of the PDGF-BB-induced migration (139% amplification without H89 to 88% amplification with H89, p = 0.02) (Fig. 3A).

To further clarify the role of protein kinase A activated by incretin in the amplification of MC3T3-E1 cell migration by PDGF-BB, we examined the effect of H89 on the GLP-1-enhanced migration by PDGF-BB. The enhancement by GLP-1 of the PDGF-BB-induced migration of MC3T3-E1 cells was significantly downregulated by H89 (292% amplification without H89 to 177% amplification with H89, p = 0.03) (Fig. 3B). We found that H89 actually attenuated the GLP-1-induced phosphorylation of CREB in these cells (Fig. 3C).

### Effects of PD98059, SP600125 or SB203580 on the amplification by GLP-1 of the PDGF-BB-induced migration of MC3T3-E1 cells and NHOsts

**Figure 4:**
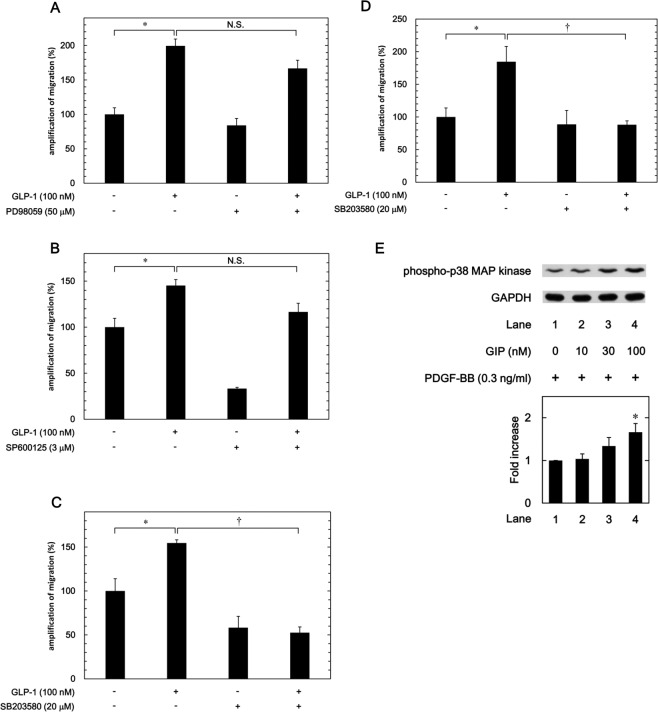
Effects of PD98059, SP600125 or SB203580 on the amplification by GLP-1 of the PDGF-BB-induced migration of MC3T3-E1 cells and NHOsts and that of GIP on the PDGF-BB-induced phosphorylation of p38 MAP kinase in MC3T3-E1 cells. MC3T3-E1 cells were incubated with 50 μM of PD98059 (**A**), 3 μM of SP600125 (**B**), 20 μM of SB203580 (**C**) or vehicle for 60 min and subsequently pretreated with 100 nM of GLP-1 or vehicle for 60 min. The cells were then stimulated by 0.2 ng/ml of PDGF-BB or vehicle for 9 h. The area of the migrated cells was measured and the PDGF-BB-induced increase in the filled area without GLP-1 was presented as 100%. *p < 0.05 compared to the value without GLP-1. ^†^*p* < 0.05 com*p*ared to the value without SB203580. N.S. indicates no significant difference between the indicated pairs. (**D**) NHOsts were incubated with 20 μM of SB203580 for 60 min and subsequently pretreated with 100 nM of GLP-1 or vehicle for 60 min. The cells were then stimulated by 0.3 ng/ml of PDGF-BB or vehicle for 6 h. The area of the migrated cells was measured and the PDGF-BB-induced increase of filled area without GLP-1 was presented as 100%. *p < 0.05 compared to the value without GLP-1. ^†^*p* < 0.05 compared to the value without SB203580. (**E**) MC3T3-E1 cells were pretreated with various doses of GIP for 60 min and then stimulated by 0.3 ng/ml of PDGF-BB for 3 min. Western blot analyses were performed using antibodies against phospho-specific p38 MAP kinase or GAPDH. The histogram shows the quantitative representation of the PDGF-BB-induced phosphorylation obtained from a laser densitometric analysis of three independent experiments. *p < 0.05 compared to the value of PDGF-BB alone.

We previously demonstrated that PDGF-BB induces the migration of MC3T3-E1 cells via p44/p42 MAP kinase, SAPK/JNK and p38 MAP kinase^12^. To further investigate whether incretin strengthens the PDGF-BB-induced migration of MC3T3-E1 cells via the activation of p44/p42 MAP kinase, SAPK/JNK or p38 MAP kinase, we examined the effects of PD98059, an inhibitor of the upstream kinase activating p44/p42 MAP kinase (MEK1/2)^33^; SP600125, an inhibitor of SAPK/JNK^34^; and SB203580, an inhibitor of p38 MAP kinase^35^ on the amplification by GLP-1 of the PDGF-BB-induced migration. Neither PD98059 nor SP600125 suppressed the enhancement by GLP-1 of the PDGF-BB-induced MC3T3-E1 cell migration (200% amplification without PD98059 to 167% amplification with PD98059, p = 0.10; 145% amplification without SP600125 to 117% amplification with SP600125, p = 0.07) (Fig. 4A,B). However, SB203580 suppressed the enhancement by GLP-1 of the PDGF-BB-induced MC3T3-E1 cell migration almost to the level of PDGF-BB-stimulation with SB203580 (155% amplification without SB203580 to 53% amplification with SB203580, p = 0.0002), (Fig. 4C). In addition, SB203580 significantly reduced the amplification by GLP-1 of the PDGF-BB-induced migration of NHOsts (185% amplification without SB203580 to 88% amplification with SB203580, p = 0.02) (Fig. 4D). Furthermore, we found that the PDGF-BB-induced phosphorylation of p38 MAP kinase was significantly augmented by GIP in MC3T3-E1 cells (Fig. 4E). We had already confirmed that no enhancement of phosphorylation of p38 MAP kinase was observed by PDGF-BB alone in osteoblast-like MC3T3-E1 cells (Supporting Information).

### Effects of systemic exendin-4 administration on the expression and localization of Rho A in osteoblasts at epiphyseal lines of the developing mouse femurs

Finally, we performed an *in vivo* assay to investigate possible changes in Rho A immunoreactivity in osteoblasts in response to incretin. We observed epiphyseal lines of P10 developing mouse femurs where clusters of osteoblasts covered the surface of bone spicules. Rho A is a major small G protein regulating the cellular movement and migration through cytoskeletal reorganization^21^. Mice with or without the intraperitoneal systemic administration of exendin-4^28^ were fixed, and frozen sections were immunostained with an antibody against osteocalcin, an osteoblast marker protein, or anti-Rho A antibody.

**Figure 5:**
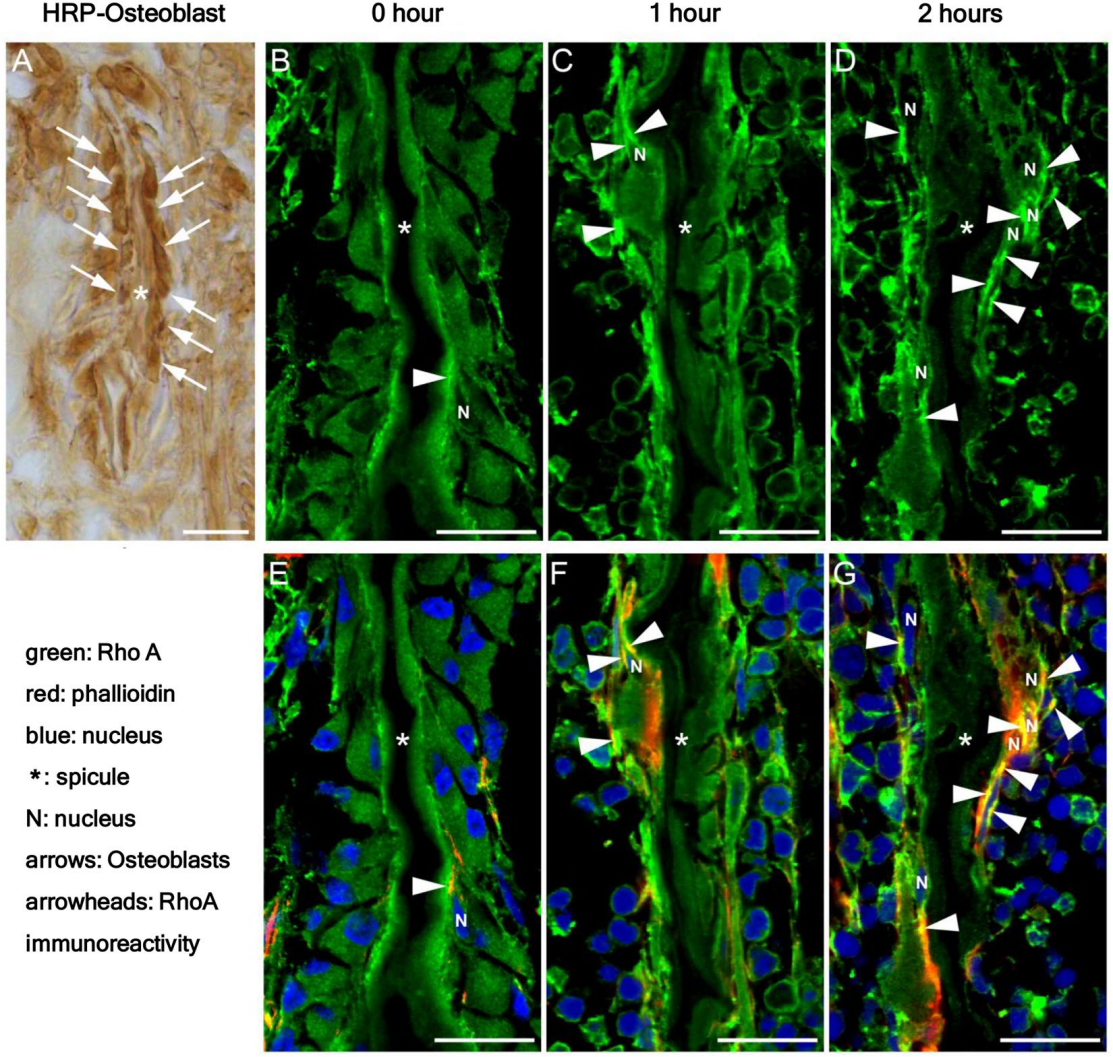
Effects of systemic exendin-4 administration on the expression and localization of Rho A in osteoblasts at epiphyseal lines of the developing mouse femurs. Cryostat sections stained by anti-osteocalcin antibody, an osteoblast marker protein (**A**, HRP-reaction product), anti-Rho A antibody (**B**–**D**) or triply stained (**E**–**G**; Rho A, green; phalloidin, red; DRAQ5, blue). The surfaces of bone spicules (*) were covered by osteocalcin-positive cells (arrows), osteoblasts (**A**). The asterisks in (**A**–**G**) indicate spicules. N in (**B**–**G**) indicate nuclei. Scale bar, 20 μm in (**A**–**G**). The overlapped area of Rho A and actin-filaments on the surface of the osteoblasts is indicated by arrowheads.

We first confirmed that the bone spicules at the epiphyseal lines were almost completely covered with osteoblast marker protein-positive cells (Fig. 5A). In osteoblasts on the surface of bone spicules, a moderate level of Rho A expression was demonstrated both in the cytoplasm and on the cell surface without exendin-4 administration (Fig. 5B). One hour after exendin-4 administration, a higher level of Rho A expression was localized on the cell surface of the small population of osteoblasts in addition to its moderate level of expression in the cytoplasm (Fig. 5C). Two hours after exendin-4 administration, high a level of Rho A expression was localized both on the cell surface and in the cytoplasm of the larger population of osteoblasts (Fig. 5D). Triple immunostaining with Rho A, actin filaments and nuclei further supported the fact that the area of Rho A accumulation at the cell surfaces, which was partially overlapped with that of actin-filaments, and demonstrated that the overlapped area of Rho A and actin-filaments on the surface of the osteoblasts was increased at the time points one and two hours after exendin-4 administration compared with the control before its administration (Fig. 5E,F,G). These data indicate that exendin-4 enhanced the Rho A expression and induced its translocation from the cytoplasm to the plasma membrane in osteoblasts on bone spicules at the epiphyseal lines of P10 developing mouse femurs.

## Discussion

In the present study, we demonstrated that the PDGF-BB-induced migration of clonal osteoblast-like MC3T3-E1 cells was time- and dose-dependently enhanced by GLP-1 and GIP that on its own had no effects on the migration.

It is firmly established that incretins released from gut enteroendocine cells after oral food ingestion act as hormones to enhance insulin secretion from β cells and suppress glucagon secretion from α cells in the pancreas^13,14,36^. Incretin receptor-activating agents, including GLP-1 receptor agonists and dipeptidyl peptidase-IV inhibitors, are currently being clinically used in the treatment of type 2 diabetes mellitus. Accumulating evidence has shown that incretins affect the cell functions of mesenchymal cells, including osteoblasts and adipocytes, in addition to pancreatic cells^15,16^. It has been shown that osteoblasts express functional receptors for GLP-1 and GIP^17,37^. With regard to the effects of incretin on the bone metabolism, GIP reportedly stimulates the differentiation of osteoblasts^17^. In addition, it has been shown that GLP-1 inhibits bone resorption by osteoclasts via calcitonin secreted from the parafollicular cells of thyroid gland^38^. Based on these findings, it is currently speculated that incretin may affect the balance between osteoblasts and osteoclasts, directing bone metabolism toward the upregulation of bone formation. To our knowledge in retrieval, this report is the first showing that incretin, which alone fails to affect osteoblast migration, accelerates the migration of osteoblasts induced by PDGF.

It is generally recognized that a main intracellular signaling path of incretin is the adenylyl cyclase/cAMP/protein kinase A pathway, and CREB is the first transcriptional regulator targeted by that pathway^13,29^. We previously confirmed that GLP-1 activates the cAMP/protein kinase A/CREB pathway in osteoblast-like MC3T3-E1 cells^30^. We therefore next investigated whether or not the cAMP/protein kinase A pathway is implicated in the enhancement by incretin of the PDGF-BB-induced migration of osteoblasts. We showed that 8-bromo cAMP and Bt_2_cAMP, cell-permeable analogues of cAMP^31^, markedly enhanced the PDGF-BB-induced MC3T3-E1 cell migration, similar to GLP-1 and GIP. In addition, H89, an inhibitor of protein kinase A^32^ that attenuated the GLP-1-induced phosphorylation of CREB in MC3T3-E1 cells, significantly suppressed the amplification by GLP-1 as well as the cAMP analogues of PDGF-BB-induced migration. Similar to our findings with MC3T3-E1 cells, we found that the PDGF-BB-induced migration of NHOsts was also accelerated by GLP-1 and that 8-bromo cAMP significantly up-regulated the PDGF-BB-induced migration. Given our present findings, the acceleration by incretins of the PDGF-BB-induced osteoblast migration is likely exerted through the adenylyl cyclase/cAMP/protein kinase A pathway.

There are some concerns about off-target kinase inhibition by H89 in the effect on the suppression of GLP-1 effect. It has been reported that H89 at 10 μM inhibits several kinases other than PKA, including S6 kinase, Rho-associated kinase and AMP-activating protein kinase^39^. Although we used H89 at 7 μM in the present study, which is lower than the above-mentioned concentration, off-target kinases inhibition by H89 might be involved in the suppression by H89 of the GLP-1 effect.

We further investigated the exact mechanism underlying the augmentation by incretin of the PDGF-BB-induced migration of osteoblast-like MC3T3-E1 cells. Regarding the intracellular signaling in the PDGF-BB-induced migration of osteoblasts, we previously demonstrated that p44/p42 MAP kinase, p38 MAP kinase and SAPK/JNK act as positive regulators in the PDGF-BB-induced migration of osteoblast-like MC3T3-E1 cells^12^. Interestingly, we showed in the present study that SB203580, an inhibitor of p38 MAP kinase^35^, but not PD98059, an inhibitor of MEK1/2^33^, or SP600125, an inhibitor of SAPK/JNK^34^, almost completely reduced the augmentation by GLP-1 of the PDGF-BB-induced MC3T3-E1 cell migration. SB203580 also reduced the amplification by GLP-1 of the PDGF-BB-induced migration of NHOsts. Therefore, our findings suggest that incretin enhances the PDGF-BB-induced migration of osteoblasts via p38 MAP kinase. Furthermore, we showed that GIP strengthened the PDGF-BB-induced phosphorylation of p38 MAP kinase in osteoblast-like MC3T3-E1 cells. Based on our findings as a whole, it is most likely that incretins accelerate the PDGF-BB-induced migration of osteoblasts through the upregulation of p38 MAP kinase activity.

We also investigated the effect of exendin-4, a GLP-1 analogue^28^, on osteoblasts *in vivo* and found that exendin-4 enhanced the expression Rho A and its translocation from the cytoplasm to the plasma membranes. Because Rho A acts as the major regulator of cellular migration by controlling actin filament organization, GLP-1 may potentiate the migration activity of osteoblasts through Rho A activation.

The osteoblast migration to the osteoclast-resorbed sites is essential for the bone remodeling process, and the migrated osteoblasts subsequently start to form bone not only during physiological bone metabolism but also in pathological states, such as repair from bone fracture and metastasis of bone tumor^3–5^. Adequate migration of osteoblasts is important for maintaining both the quality and quantity of bone, and the impairment of osteoblast migration may deteriorate the bone strength, resulting in an increased risk of fracture. It is well established that diabetes mellitus is accompanied with an increased risk of osteoporotic fracture^40^. In the present study, we clearly demonstrated that incretin in collaboration with PDGF-BB accelerates osteoblast migration. Taken together, these findings suggest that incretin related agents, including GLP-1 analogues, are useful therapeutic tools for type 2 diabetes mellitus and may ameliorate the risk of bone fracture in these patients.

Our findings may also support the importance of oral food ingestion-inducible physiological incretin secretion in bone health. The incretin released after meal intake may possess a preventive effect for osteoporosis and accelerate fracture healing especially in elderly people. Further investigations will be required in order to clarify the exact mechanism underlying the function of incretin in osteoblast migration and bone metabolism.

## Conclusion

Taken together, our results strongly suggest that incretin accelerates the PDGF-BB-induced migration of osteoblasts via protein kinase A, and that the amplification by incretin is mediated at least in part via p38 MAP kinase activation. The findings from this study may provide a great potential for incretin in bone physiology and therapeutic strategy against bone repair and osteoporosis.

## Supplementary information

Supplementary Figures.
