# Supplementary material for: Incretin accelerates platelet-derived growth factor-BB-induced osteoblast migration via protein kinase A: The upregulation of p38 MAP kinase
Source: Sci Rep. 2020 Feb 11;10:2341. doi: 10.1038/s41598-020-59392-7 (PMC7012849; doi:10.1038/s41598-020-59392-7)
Supplement: Supplementary file 1 — Supplementary Figures. [file 41598_2020_59392_MOESM1_ESM.pdf]

Supporting Information for

Incretin accelerates platelet-derived growth factor-BB-induced osteoblast migration via protein kinase A: upregulation of p38 MAP kinase

Tetsu Kawabata[1,3,5], Haruhiko Tokuda[1,6], Gen Kuroyanagi[1,4], Kazuhiko Fujita[1,3], Go Sakai[1,3], Woo Kim[1,2], Rie Matsushima-Nishiwaki[1], Hiroki Iida[2], Ken-ichiro Yata[7], Shujie Wang[8], Akira Mizoguchi[8], Takanobu Otsuka[3] and Osamu Kozawa[1,*]

Departments of [1]Pharmacology, and [2]Anesthesiology and Pain medicine, Gifu University Graduate School of Medicine, Gifu 501-1194, Japan;

Departments of [3]Orthopedic Surgery, and [4]Rehabilitation Medicine, Nagoya City University Graduate School of Medical Sciences, Nagoya 467-8601, Japan;

[5]Department of Orthopedic Surgery, Toyokawa City Hospital, Toyokawa 442-8561, Japan; [6]Department of Clinical Laboratory/ Medical Genome Center Biobank, National Center for Geriatrics and Gerontology, Obu 474-8511, Japan;

Departments of [7]Neurology, and [8]Neural Regeneration and Cell Communication, Graduate School of Medicine, Mie University, Tsu 514-8507, Japan.

[*]Corresponding author

Email: okozawa@gifu-u.ac.jp (O. Kozawa)

Address: 1-1 Yanagido, Gifu 501-1194, Japan

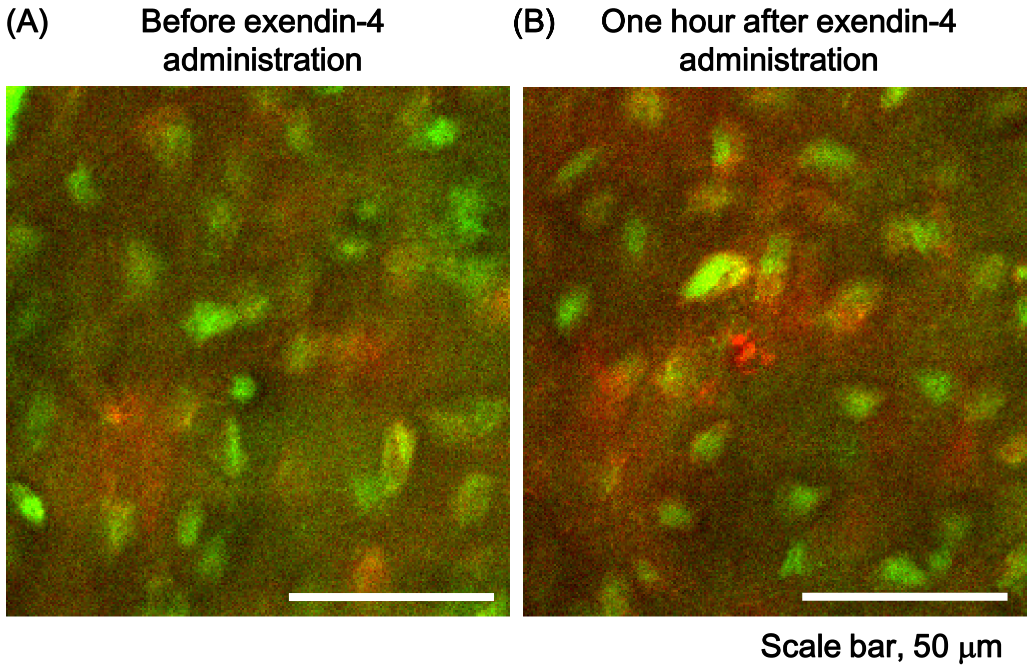

Fig. S1 Multiphoton laser microscopy images of adult mouse cranial bones of green mice. (A) Before exendin-4 administration; (B) One hour after exendin-4 administration in the cranial bone.

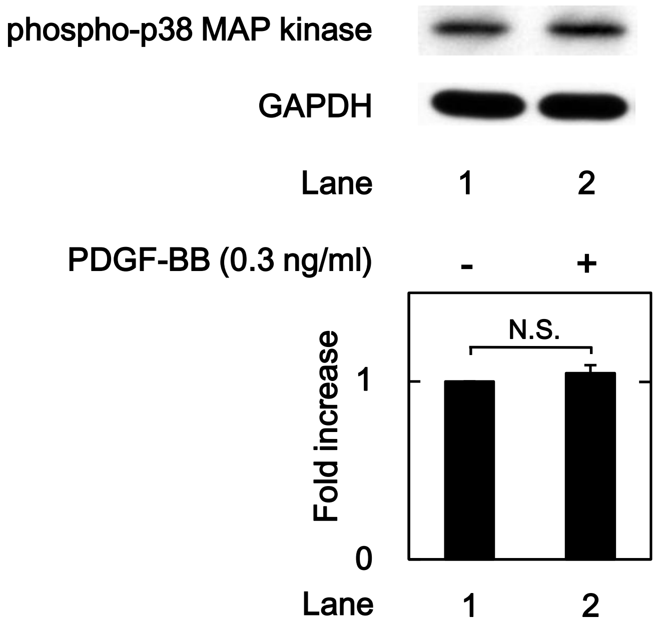

Fig. S2 Effect of PDGF-BB (0.3 ng/ml) on the phosphorylation of p38 MAP kinase in MC3T3-E1 cells. MC3T3-E1 cells were stimulated by 0.3 ng/ml of PDGF-BB or vehicle for 3 min. Western blot analyses were performed using antibodies against phospho-specific p38 MAP kinase or GAPDH. The histogram shows the quantitative representation of the PDGF-BB-induced phosphorylation obtained from a laser densitometric analysis of three independent experiments. N.S. indicates no significant difference between the indicated pairs.
